# Meigs syndrome presenting with recurrent unilateral pleural effusion

**DOI:** 10.1002/rcr2.1421

**Published:** 2024-06-26

**Authors:** Boon Hau Ng, Sarah Hani Johari How, Nik Nuratiqah Nik Abeed, Hsueh Jing Low, Rose Azzlinda Osman, Andrea Ban Yu‐Lin

**Affiliations:** ^1^ Respiratory Unit, Department of Medicine, Faculty of Medicine Universiti Kebangsaan Malaysia, Hospital Canselor Tuanku Muhriz Kuala Lumpur Malaysia; ^2^ Department of Anaesthesia and Critical Care, Faculty of Medicine Universiti Kebangsaan Malaysia, Hospital Canselor Tuanku Muhriz Kuala Lumpur Malaysia

**Keywords:** ovarian fibroma, pleural effusion, salphingo oophorectomy

## Abstract

Pelvic tumours are a rare cause of pleural effusion. We describe an approach to a case of Meigs syndrome with recurrent unilateral pleural effusion. A woman in her 60s' presented with recurrent right‐sided pleural effusion, leading to cough and shortness of breath. Thoracentesis yielded exudative pleural fluid with cytology negative for malignancy. Pleuroscopy revealed inflamed pleura, and pleural biopsy was consistent with inflammatory changes. The patient's cancer antigen 125 level was elevated at 256 U/mL. Given the high suspicion of malignancy, a computed tomography scan of the chest, abdomen, and pelvis was performed and revealed ascites and a large left ovarian and uterine mass. The patient underwent a total abdominal hysterectomy and bilateral salphingo oophorectomy after experiencing three additional episodes of pleural effusion. Histological examination revealed the left ovarian mass to be a cellular fibroma and the uterine masses to be leiomyomata. Following the operation, there was no recurrence of pleural effusion.

## Graphical abstract

This case illustrates Meigs syndrome secondary to an ovarian fibroma, which resulted in recurrent pleural effusion. A thorough workup and pleural biopsy excluded other potential pathologies.

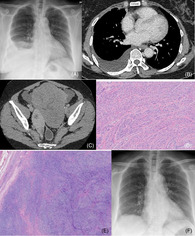

## INTRODUCTION

Recurrent pleural effusion in patients with a pelvic mass and elevated CA125 levels often presents a diagnostic challenge for physicians, as it can closely resemble malignant pleural effusion. Nevertheless, surgery and histological examination are essential to confirm the preoperative diagnosis, as patients with these symptoms may have a benign condition known as Meigs syndrome. Meigs syndrome is named after Joe Vincent Meigs, who in 1937 described benign ovarian fibromas or fibroma‐like tumours that are associated with ascites and pleural effusion, which resolve following the removal of the tumour.^1^ We present a case of Meigs' syndrome with recurrent pleural effusion, where thorough workup and pleural biopsy excluded other pathologies. This case includes a discussion of clinical findings and a review of the literature on this rare condition.

## CASE REPORT

**FIGURE 1:**
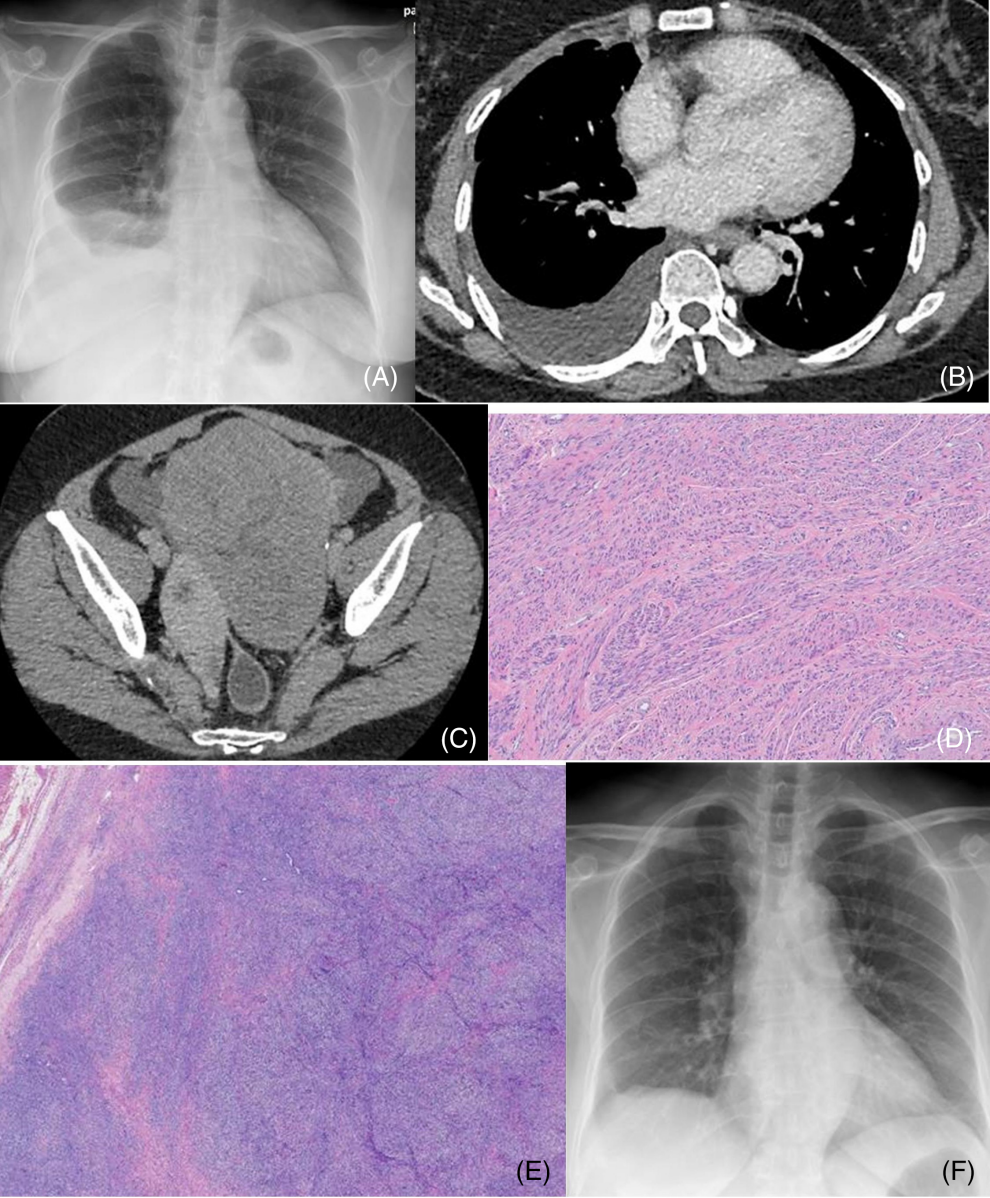
(A) Chest radiograph demonstrates right‐sided pleural effusion. (B) CT of the chest demonstrates right‐sided pleural effusion. (C) CT of the abdomen and pelvis demonstrates heterogenous hypodense mass arising from the uterus. (D) The uterine tumours were composed of intersecting fascicles of neoplastic and benign smooth muscle cells ((leiomyoma). (E) Histological examination of the ovarian mass shows a circumscribed and solid lesion composed of intersecting fascicles of spindle cells (fibroma). (F) A chest radiograph demonstrates complete resolution of pleural effusion at post‐operative follow‐up.

A woman in her 60s' presented with recurring right pleural effusion. She complained of intermittent cough and shortness of breath. Her vital signs were stable, with a blood pressure of 122/82 mmHg, heart rate of 78 beats per minute, respiratory rate of 26 breaths per minute, temperature of 37°C, and oxygen saturation of 95% on room air. Physical findings included reduced breath sounds, dullness on percussion, decreased vocal resonance over the right hemithorax, and ascites. There was a vague abdominal mass palpable at the suprapubic region. Bedside thoracic ultrasonography revealed a hypoechoic effusion spanning three rib spaces at the right hemithorax. The chest radiograph showed a moderate right pleural effusion (Figure 1A).

Thoracentesis exhibited exudative characteristics, leading to the insertion of a chest tube using the Seldinger technique. Autoimmune panel investigations, including antinuclear antibody and anti‐double‐stranded DNA, returned negative results, with normal complement 3 and complement 4 levels. Echocardiogram results showed a 57% ejection fraction without regional wall motion abnormalities. Elevated CA 125 levels at 256 U/mL were noted.

Computed tomography scan of the thorax, abdomen and pelvis revealed a heterogeneous hypodense mass originating from the uterus's left lateral and posterior aspect, ovarian mass, ascites and right moderate pleural effusion (Figure 1B,C). Pleuroscopy was performed which revealed nonspecific inflamed costoparietal pleura. A pleural biopsy showed fibro‐collagenous tissue lined by mesothelial cells consistent with acute‐on‐chronic inflammation. Pleural biopsy for mycobacterium tuberculosis GeneXpert and cultures were negative. Based on the CT findings and the elevated CA 125 levels, the working diagnosis was ovarian carcinoma. She was advised to undergo a total abdominal hysterectomy and bilateral salphingo oophorectomy, which she declined.

She was followed up at the chest clinic and experienced three further recurrences of symptomatic pleural effusion over a two‐year period. During each visit, she required chest drain insertions via the Seldinger technique to remove 2–2.5 L of serous effusion. We did not perform any pleurodesis in this patient due to the unresolved nature of the diagnosis. The successive biochemical analysis of the pleural fluid was exudative in nature. After the third chest tube insertion, she finally agreed to undergo a total hysterectomy and bilateral salphingo oophorectomy with omentectomy which revealed a cellular fibroma of the left ovary and uterine leiomyomata on histopathological examination (Figure 1D,E). The patient was followed up at the respiratory clinic at 1 month, 3 months, and 6 months post‐surgery. No clinical or radiological signs of recurrence of right pleural effusion were observed during these follow‐up visits (Figure 1F).

## DISCUSSION

The manifestation of pleural effusion is frequently linked with malignant tumours, nephrotic syndrome, liver cirrhosis, congestive cardiac failure, and tuberculosis.^2^ Individuals presenting with unexplained unilateral pleural effusion commonly undergo diagnostic thoracentesis and are referred to a respiratory physician. Meigs syndrome is estimated to occur in about 1% of ovarian tumours.^2^ Moreover, pleural effusion is observed in approximately 1% of Meigs syndrome cases. The definitive diagnosis of Meigs syndrome is typically confirmed postoperatively with evidence of resolution of pleural effusion and ascites, along with confirmation through tissue diagnosis.

In countries where tuberculosis is endemic, such as Malaysia, pleural effusion remains a diagnosis of high suspicion that requires thorough investigation to rule out tuberculosis. Pleural effusion samples often have a low yield for acid‐fast bacilli stains and cultures, so negative results do not rule out tuberculosis.^3^ Pleural biopsy, culture of pleural tissue, and elevated pleural fluid adenosine deaminase (ADA) levels are often useful in confirming the diagnosis of tuberculosis. In our case, recurrent pleural effusion was unlikely to be caused by tuberculosis due to negative pleural tissues for mycobacterial cultures, histopathological findings and a low level of pleural fluid ADA (6.0 U/L).

The pathophysiology of pleural effusion in Meigs syndrome remains uncertain. Proposed mechanisms include direct pressure on surrounding lymphatics or vessels, hormonal stimulation, and fluid transudation from the tumour surface. Most cases report exudative right pleural effusion, likely due to seepage of ascitic fluid through minute diaphragmatic defects, commonly seen on the right due to the anatomical location of the inferior vena cava opening.^4^ Up to 70% of pleural effusions are right‐sided, while 15% are left‐sided and bilateral, respectively.^3^

An elevated CA‐125 level lacks specificity and can manifest in both gynaecological and nongynecological conditions. Research indicates a positive correlation between CA‐125 concentration and the volume of pleural effusion.^5^ A study on 30 patients with different aetiology of effusions showed pleural CA‐125 to be higher in malignant pleural effusions and also higher in tuberculosis compared to other infections. High levels of CA‐125 have been found in some cases of Meigs syndrome (Table 1). The presence of an ovarian mass accompanied by elevated levels of cancer antigen 125 (CA‐125) should raise suspicion for malignancy.

**TABLE 1:** Literature review.

Author/year	Age	Presentation	Ca‐125 level (<35 U/mL)	PF side/nature	HPE diagnosis	Follow‐up/outcome
Hou et al./2021^4^	52	Dry cough	663.3 U/mL	Right/Transudative	Ovarian thecoma	12 weeks post‐op no recurrence of symptoms
Riker et al./2013^6^	54	Dyspnea, abdominal distension	1191 U/mL	Bilateral/exudative	Ovarian fibroma	3 months post‐op, no recurrence of symptoms
Fujiwara et al./2018^7^	50	Dyspnoea, abdominal distension	1237 U/mL	Right/−	Struma ovarii	6 months post‐op, no recurrence of symptoms
Pauls et al./2019^8^	56	Dyspnea	437 U/mL	Right/exudative	Hydropic leiomyoma	8 months post‐op, no recurrence of symptoms
Khanduja et al./2021^9^	50	Dyspnoea, dry cough	234 U/mL	Right/exudative	Ovarian fibroma	Few months post‐op no recurrence of symptoms
Tan et al./2022^10^	46	Abdominal distension	56.5 U/mL	Bilateral/−	Ovarian fibroma	Symptoms resolved temporarily but recurred due to infection post‐op
Patel et al./2022^11^	55	Dyspnoea, abdominal distension	425 U/mL	Right/exudative	Struma ovarii	8‐weeks post‐op, no recurrence of symptoms

Abbreviations: HPE, histopathological examination; L, left; PF, pleural effusion; post‐op, post‐operatively; R, right; TAHBSO, total abdominal hysterectomy and bilateral salphingo oophorectomy.

This case describes a patient with ovarian fibroma and incidental uterine leiomyomata presenting with recurrent unilateral pleural effusion, ascites, and elevated CA‐125 levels, mimicking symptoms of pelvic malignancy. However, the pleural effusion and ascites ultimately resolved following the total hysterectomy and bilateral salphingo oophorectomy. It underscores the importance of maintaining a broad range of differential diagnoses when managing similar cases, as definitive diagnosis typically occurs only after surgery.

## CONFLICT OF INTEREST STATEMENT

Dr Andrea Ban Yu‐Lin is an Editorial Board member of Respirology Case Reports and a co‐author of this article. She was excluded from all editorial decision‐making related to the acceptance of this article for publication.

## ETHICS STATEMENT

The authors declare that appropriate written informed consent was obtained for the publication of this manuscript and accompanying images.

## Data Availability

The data that support the findings of this study are available on request from the corresponding author. The data are not publicly available due to privacy or ethical restrictions.
